# The Composition of Nitrogen-Fixing Microorganisms Correlates With Soil Nitrogen Content During Reforestation: A Comparison Between Legume and Non-legume Plantations

**DOI:** 10.3389/fmicb.2019.00508

**Published:** 2019-03-14

**Authors:** Jie Chen, Weijun Shen, Han Xu, Yide Li, Tushou Luo

**Affiliations:** ^1^Research Institute of Tropical Forestry, Chinese Academy of Forestry, Guangzhou, China; ^2^Key Laboratory of Vegetation Restoration and Management of Degraded Ecosystems, South China Botanical Garden, Chinese Academy of Sciences, Guangzhou, China; ^3^Research Institute of Subtropical Forestry, Chinese Academy of Forestry, Hangzhou, China

**Keywords:** nitrogen-fixation, forest restoration, subtropical forest, legume, *nifH* gene

## Abstract

Numerous reforestation projects have been conducted to improve soil fertility in degraded forests, often causing alterations to the soil microbial communities. However, it remains unclear whether microbial functional groups are affected and how these groups correlate with an increase in the nutrient contents during reforestation. We investigated the abundance and composition of free-living nitrogen-fixing microorganisms (diazotrophs) by quantifying and sequencing the marker gene *nifH* in bulk soils from five reforestation approaches, including legumes and non-legumes, in subtropical China. The relationships between diazotrophic community attributes and soil nitrogen (N) content [NO_3_^−^, NH_4_^+^, and microbial biomass N (MBN)] were examined under various approaches. Abundance of diazotrophs was highest in the native tree plantation (*Schima* spp. and *Michelia macclurei*) and *Acacia mangium* monoculture (AM), and lowest in the *Pinus massoniana* monoculture. The diazotrophic abundance correlated positively with soil organic matter and water content while there was a negative correlation to pH. The composition of diazotrophic community differed significantly among the five reforestation approaches examined and was closely correlated with variations in soil pH, NH_4_^+^ and water content. Diazotrophic community composition was closely related to soil NH_4_^+^ content, whereas abundance was not. The AM contained higher NH_4_^+^, NO_3_^−^ and MBN contents than the other reforestation approaches, which may be associated with the indicator species of diazotrophs (*Actinobacteria*, *Proteobacteria*, and *Firmicutes*). However, there were more indicator species of *Proteobacteria* in the mixed *Acacia* plantation (*Acacia mangium* and *Acacia crassicarpa*) than in AM, which might have contributed to the remarkedly lower N content compared to AM. Overall, the soil N content under reforestation appeared to be more related to the composition of diazotroph community than to the abundance of diazotrophs.

## Introduction

Nitrogen (N) availability in forest soils plays a significant role in regulating plant growth, microbial activity and the interactions between plant and soil microorganisms (Kaye and Hart, 1997; Dijkstra et al., 2008). To maintain biodiversity and function of forest ecosystem, an adequate N input is needed. Unlike agricultural lands that receive N from fertilization, forests usually gain N from natural inputs, such as atmospheric N deposition, organic N decomposition and biological N fixation (BNF) (Mirza et al., 2014). The latter is the dominant form of N input in forest ecosystems, accounting for as much as 97% (Galloway et al., 2008). A recent study demonstrated different effects of N enrichment on soil organisms, carbon (C), and N mineralization with and without plantation in a semi-arid grassland (Chen et al., 2018). Therefore, vegetation induced indirect N inputs may have considerable effects on the functioning of soil ecosystem.

Biological N fixation is driven by a highly diverse group of microorganisms (Dixon and Kahn, 2004). All N-fixing microorganisms harbor the same functional gene *nifH*, which encodes one sub-unit of the nitrogenase enzyme and is widely used as a marker for studying the abundance and composition of N-fixers in various environments. Mirza et al. (2014) investigated the *nifH* gene and found that forest-to-pasture conversion in the Amazon rainforest altered the diazotrophic composition rather than their abundance. In Alaskan boreal soil, Penton et al. (2016) used *nifH* sequencing data to determine that the N-fixer composition in a permafrost thaw gradient varied with the depth of the water table. Previous studies mainly focused on free-living N-fixers (diazotrophs), although symbiotic N-fixers are conventionally recognized as the main drivers of BNF (Gutschick, 1981). Therefore, it is imperative that the features and determinants of free-living diazotrophs in different environments are evaluated to determine the responses of soil N to environmental changes and the affecting factors.

The decline in soil fertility caused by forest degradation is a severe ecological problem globally. In forest ecosystems, the soil N content is sensitive to deforestation and soil erosion (Atucha et al., 2013). Deforestation can decrease soil organic N by reducing litter input and root exudates. Moreover, large amounts of plant biomass N cannot return to soil after deforestation but is emitted into atmosphere as N oxides when wood is burned as fuel (Silva et al., 2011). Reforestation is important to control soil degradation and improve soil fertility (Albert, 2015). The effects of reforestation on soil N generally vary with the plant species (Yuan et al., 2012; Wang et al., 2013). To gain N quickly, leguminous species are introduced to degraded land. Wang et al. (2010) compared the soil properties and N transformations between N-fixing and non-N-fixing plantations, and found that N-fixing plants were more effective in recovering N cycling processes in degraded subtropical forests. Franco and Faria (1997) discussed the importance of leguminous trees in land reclamation and sustainability of the tropical systems reporting that these trees increased the soil N stock by 0.19 Mg ha^−1^ year^−1^ in tropical forests, which promoted successful revegetation of degraded land. However, whether the increased soil N supply after planting legumes correlates with changes in the diazotrophic community abundance and composition remains unclear. Some researchers argue that planting local dominant species is more important in recovering the ecosystem’s function (Mcnamara et al., 2006; Hayden et al., 2010; Lu et al., 2017), while others demonstrated that the natural recovery of vegetation without human disturbance was an effective approach for soil nutrient sequestration (Zheng et al., 2004). However, limited studies have associated the re-establishment of soil nutrient content with alterations in specific microbial functional groups during reforestation.

The composition and abundance of the diazotrophic community in soil could be correlated with several factors, including soil N, C and phosphorus contents, soil texture, pH, soil temperature and moisture (Limmer and Drake, 1996, 1998; Burgmann et al., 2005; Hsu and Buckley, 2009). Moreover, the correlations between diazotrophic community and environmental factors often differ among vegetation types. Soil physicochemical properties could be modified by specific plant species through litter-fall, root phenes, and exudates containing different nutrients, which subsequently influence the microenvironment of the diazotrophic community (Saleem et al., 2018). The different N requirements of specific plant species may be an underlying driver of the diazotrophic community. The specific abundance and composition of diazotrophic community and their associations with soil N availability under different tree species are largely unknown. This limits our ability to evaluate the effectiveness of reforestation using various tree species.

In this study, we investigated the responses of the diazotrophic community in five reforestation approaches in subtropical China. One approach was natural revegetation (NR), and the other four were artificial plantations of different tree species, including a non-native legume monoculture of *Acacia mangium* (AM), non-native mixed legumes (ML; *Acacia mangium* and *Acacia crassicarpa*), native dominant species (NP; *Schima* spp. and *Michelia macclurei*) and native *Pinus massoniana* monoculture (PM). The abundance and composition of diazotrophs in growing season were determined using real-time PCR and amplicon sequencing targeting *nifH*. Correlations between soil physicochemical properties with abundance and composition of diazotrophic community were examined. Our objectives were to (1) clarify the abundance and composition of the diazotrophic community under the five reforestation approaches and their correlations with soil N content, and (2) analyze the predominant soil properties that relates to the abundance and composition of the diazotrophs under reforestation.

## Materials and Methods

### Study Site

This study was conducted at the Heshan National Field Research Station of Forest Ecosystem (112°50′E, 22°34′N), Guangdong Province, South China. This region has a subtropical monsoon climate, with mean annual precipitation of 1,668 mm and temperature of 22.5°C. The soil is classified as laterite (Oxisol in the USDA soil taxonomy), developed from sandstone. The original forest type is evergreen broadleaf with a large area severely disturbed owing to anthropogenic activities and deforestation, leading to soil erosion and degradation. Five reforestation approaches have been conducted in the degraded region since 2005. One approach was NR, and the other four were artificial plantations of different tree species, including a non-native legume monoculture of *Acacia mangium* (AM), non-native mixed legumes (ML; *Acacia mangium* and *Acacia crassicarpa*), native dominant species (NP; *Schima* spp. and *Michelia macclurei*) and native *Pinus massoniana* monoculture (PM). The NR was dominated by *Miscanthus sinensis* and *Blechnum orientale*, and a few shrubs, such as *Rhodomyrtus tomentosa*, *Melastoma candidum*, *Gardenia jasminoides* and *Ilex asprella*. The main understory shrubs were *Limea rotundifolia*, *Aporosa dioica*, *Cinnamomum burmannii* and *Psychotria rubra* in PM; *Carallia brachiate*, *Syzygium levinei*, and *Syzygium cumini* in AM; *Ilex asprella*, *Rhodomyrtus tomentosa*, and *Dicranopteris pedata* in ML; and *Psychotria rubra*, *Aporosa dioica* and *Litsea glutinosa* in NP. Each reforestation approach had three replicated plots, randomly distributed in the experimental area. The area of each plot is 1 ha, and the total area of the experimental region is 50 ha. Trees were planted with a spacing of 2 m × 3 m in the four plantations, and tree density was ∼1,650 ha^−1^.

### Soil Sampling and Physicochemical Analysis

Soil sampling was conducted between trees to avoid the collection of rhizosphere soils. Two sampling sites were randomly selected in each plot during the growing season (August 2016), and three subsamples were collected at each site from the top layer (0–10 cm) using an anger (Φ 5 cm) and mixed thoroughly as one sample. Thus, two composite samples were collected in each plot, and a total of six composite samples were generated in each reforestation approach. These served as six replicates. Soil samples were sieved through a 2-mm mesh after removing litter, roots, and stones, and immediately taken to the laboratory in an ice box. Samples were divided into two: one was used for soil physicochemical analyses and stored at 4°C, while the other was used for the microbial analyses and stored at −20°C. Soil samples were analyzed within 2 weeks.

Soil pH was determined in a soil:water (1:2.5) suspension using a pH meter (Denver Instrument UB-7 pH/ev Meter, United States). The soil water content (SWC) was determined using the oven drying method. Soil organic matter (SOM), NH_4_^+^ and NO_3_^−^ concentrations were measured using K_2_Cr_2_O_7_ oxidation, indophenol blue colorimetry and copperized cadmium reduction methods, respectively (Liu et al., 1996). Soil dissolved organic C (DOC) was determined from the 0.5 M K_2_SO_4_ extraction using a total organic C analysis instrument (TOC-VCSH, Shimadzu, Japan). Soil microbial biomass C (MBC) and microbial biomass N (MBN) were measured using the fumigation method (Vance et al., 1987). Briefly, 10 g of fresh soil was weighed into a glass beaker and fumigated with chloroform in a vacuum glass dryer for 24 h in dark. Then, the fumigated soil was extracted with 0.5 M K_2_SO_4_, and the extracted liquid from the un-fumigated soils was used for the DOC measurement. MBC and MBN were calculated as the difference in the C and N concentration, respectively, between fumigated and non-fumigated samples, divided by 0.45 and 0.54, respectively.

### DNA Extraction and *nifH* Gene Quantification

Soil total DNA extraction and purification were performed using the HiPure Soil DNA Mini Kit (Magen, Guangzhou, China) with 0.3 g fresh soil. DNA solution was quantified using a NanoDrop 2000 spectrophotometer (Thermo Fisher Scientific Inc., United States), and the DNA solution was stored at −20°C for further analyses.

The *nifH* gene abundance was measured using absolute real-time PCR method on an ABI 7500 thermocycler system (Applied Biosystems, Foster City, CA, United States). The absolute real-time PCR correlates the PCR signal to input copy numbers using a calibration curve, and neither comparisons nor references are needed (Pfaffl, 2004). The primers *nifH*F (AAAGGYGGWATCGGYAARTCCACCAC) and *nifH*R (TTGTTSGCSGCRTACATSGCCATCAT) were used during real-time PCR (Rösch et al., 2002). A 96-well plate was used, with each well contained 12.5 μl of SYBR Premix Ex Taq (TaKaRa Biotechnology, Japan), 1 μl of each primer (10 mmol/L) and 2 μl of DNA template (1–10 ng). The following three-step amplification protocol was used for quantification: 1 cycle at 95°C for 30 s, and 40 cycles of 5 s at 95°C, 34 s at 55°C and 1 min at 72°C. A standard curve was generated from a 10-fold serial dilution (10^3^–10^8^ copies per μl) of plasmids extracted from clones containing the target functional gene fragment. The number of gene copies was directly calculated from the extracted plasmid DNA concentration, and was presented as functional gene abundance. The PCR efficiency and correlation coefficients (*R*^2^) for standard curves were 90.12% and 0.999, respectively.

### *NifH* Gene Sequencing and Bioinformatics Analyses

The composition and diversity of diazotrophic community were detected using amplicon sequencing. Primers *nifH*F/*nifH*R tagged with multiplex identifier sequences were used in amplification of *nifH* sequences (Poly et al., 2001) with the Illumina Nextera adapter A on *nifH*F and adapter B on *nifH*R. Briefly, the PCR reaction was performed in 96-well plates (Axygen, United States) on a Bio-Rad S1000 thermal cycler (Bio-Rad Laboratory, Hercules, CA, United States), with 50-μl volumes in each well, which included 25 μl of 2× Premix Taq (TaKaRa Biotechnology, Japan), 2 μl of each primer (10 mM), 5 μl of DNA template (60 ng) and 16 μl of RNase free Ultra-Pure water. The amplification was performed under the following conditions: 5 min at 94°C; 30 cycles of 30 s at 95°C, 30 s at 55°C and 30 s at 72°C; followed by 10 min at 72°C. Each soil sample had three amplification replicates, and the PCR products from the three replicates were mixed. Then, the PCR products were pooled and purified using a PCR Purification Kit (Axygen Bio, United States) and diluted to a concentration of 10 ng ml^−1^ before sequencing. Finally, the paired-end sequencing of *nifH* amplicons was carried out using the Illumina HiSeq sequencer at Genepioneer Biotechnology Co., Ltd. (Nanjing, China).

Paired-end reads were merged with FLASH, and low quality sequences were discarded using QIIME (Caporaso et al., 2010). Chimeric composite sequences were filtered using Usearch (Edgar, 2010). Chimera-free *nifH* sequences were grouped into operational taxonomic units (OTUs) based on a threshold 97% identity level. The taxonomic classification of Chimera-free sequences was determined using the proGenomes database^1^. All *nifH* sequences obtained in this study have been deposited in GenBank under accession no. PRJNA486256.

To increase the taxonomic resolution of the *nifH* sequences (Lema et al., 2012; Mirza et al., 2014), the sequences were translated into amino acid sequences and then aligned on the functional gene pipeline (Fungene) within the Ribosomal Data Project (Cole et al., 2009). The aligned protein sequences were used for the clustering of operational protein units (OPUs) at 99%, 97%, 95%, 90%, and 80% similarity levels. A phylogenetic tree was constructed using OPUs containing more than five sequences on PHYML 3.0 with the maximum likelihood method (Guindon et al., 2005). A representative *nifH* sequence for each OPU was selected based on the relative abundance and was used for the taxonomic identification.

### Composition and Diversity of the Diazotrophic Community

Shannon-Wiener index of the diazotrophic community was calculated for samples with the OTU table to analyze the species diversity. Non-metric multidimensional scaling (NMDS) based on the Bray–Curtis similarity was used to investigate the patterns of diazotrophic community structure. Statistical significance of the differences among five reforestation approaches was tested using the PerMANOVA method with 999 permutations (Anderson, 2001). The OTU tables of *nifH* abundance and presence/absence matrix were applied when conducting NMDS. Vegetation variables were converted to dummy binary variables before conducting the structural equation model (SEM) analysis, namely, PM = 1, ML = 2, NR = 3, NP = 4, and AM = 5. The SEM analysis was conducted to clarify how vegetation type could, directly and indirectly, affect diazotroph composition by using AMOS 21.0 (SPSS Inc., Chicago, IL, United States). Goodness of the model fits were examined by chi-square test (*p* > 0.05), goodness fit index (*GFI* > 0.95), comparative fit index (*CFI* > 0.95) and root mean square errors of approximation (*RMSEA* < 0.05) (Schermelleh-Engel et al., 2003). The model containing three soil properties (NH_4_^+^, SWC, and pH) showed the best fit, with *p* = 0.54, *GFI* = 0.96, *CFI* = 1, and *RMSEA* = 0. We therefore only reported the results of this model. Venn diagrams were drawn to show the unique and common OTUs between samples (Supplementary Figure S1). To identify the indicator species in each reforestation approach, the indicator values of the diazotrophic species were calculated under each approach as the product of the relative abundance and relative frequency of occurrence (Dufrene and Legendre, 1997). As a consequence, a greater indicator value was returned when more individuals of a species were found under a single reforestation approach, and the species occurred in more sampling sites under the same reforestation approaches (Supplementary Table S1). The statistical significance of the indicator value was assessed using a 999-site randomization procedure (Edgington, 1995). Briefly, in each randomization, samples were randomly redistributed and an indicator value was returned. A 95% confidence range was obtained by calculating the 2.5 and 97.5 percentiles of the randomly generated indicator values. If the tested indicator value was outside of the 95% confidence range, then the tested value was significant. All analyses were performed on R 3.3.2 (R Core Team, 2013) using the packages “Vegan” (Oksanen et al., 2013) and “labdsv” (Roberts, 2013).

### Statistical Analyses

The normality (Kolmogorov–Smirnov test) and homogeneity of variances (Levène test) for variables were determined before statistical analyses. One-way analyses of variance with Tukey’s multiple comparisons were used to test the differences of soil physicochemical properties and diazotrophic community attributes among the five reforestation approaches. Soil physicochemical variables and the NH_4_^+^:NO_3_^−^ ratio were subjected to principal component analyses to characterize and explain the soil nutrient conditions of different reforestation approaches. Spearman correlations between soil physicochemical variables and *nifH* abundance were calculated.

The OTU table of indicator species and soil physicochemical properties were subjected to canonical correspondence analysis (CCA) to detect the determining factors of the indicator species. The best CCA model was selected based on the Akaike’s Information Criterion value. The significance of the selected CCA model and the soil properties included in the model were tested by 999 permutations (Legendre and Legendre, 2012). All analyses were performed on R 3.3.2 (R Core Team, 2013) with the package “Vegan” (Oksanen et al., 2013). The significance level for the analyses was set as *p* < 0.05.

## Results

### Soil Physicochemical Properties

**FIGURE 1:**
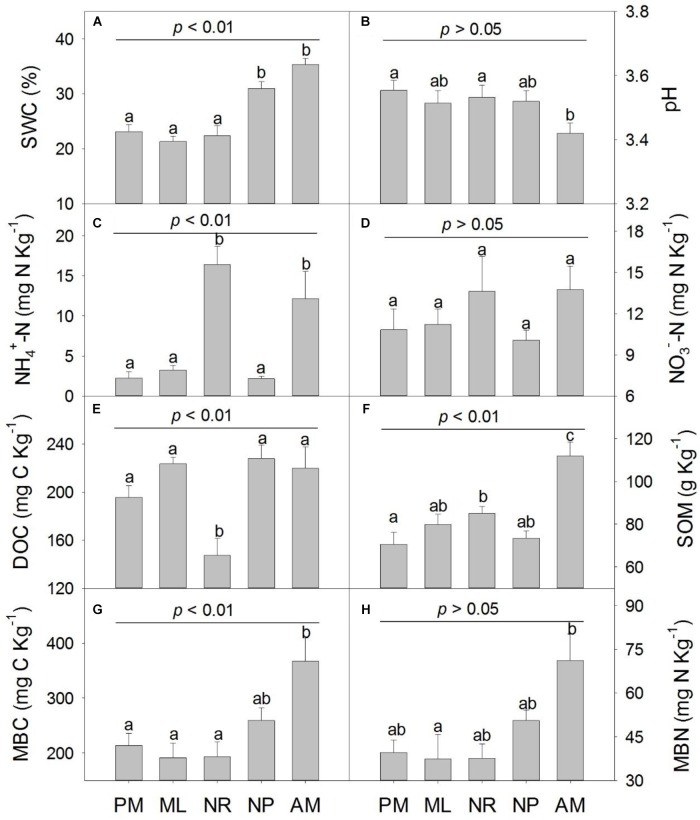
Soil physicochemical properties in five reforestation approaches in subtropical China. **(A)** soil water content (SWC), **(B)** soil pH, **(C)** soil NH_4_^+^ content, **(D)** soil NO_3_^−^ content, **(E)** soil dissolved organic carbon (DOC), **(F)** soil organic matter (SOM), **(G)** soil microbial biomass carbon (MBC) and **(H)** soil microbial biomass nitrogen (MBN). The five reforestation approaches were: *Pinus massoniana* monoculture (PM), mixed *Acacia crassicarpa* and *Acacia mangium* (ML), natural revegetation (NR), native tree plantation (NP), and *Acacia mangium* monoculture (AM). Error bars represent the standard errors of the mean (*n* = 6). The upper *p*-value in each chart represent the overall difference in the variation among five approaches; different lowercase letters indicate the significant differences of multiple comparisons at *p* ≤ 0.05.

In the five reforestation approaches, the soil properties varied significantly except for soil pH, NO_3_^−^, MBN content and MBC:MBN ratio (Figure 1 and Supplementary Figure S2B). The SWC was significantly higher in NP and AM, and soil MBC and MBN was greater in AM than in PM, ML, and NR (Figure 1G,H). The soil NH_4_^+^content and the NH_4_^+^:NO_3_^−^ ratio was significantly higher in NR and AM (Figure 1C and Supplementary Figure S2A). However, the soil DOC was considerably lower in NR than in the other reforestation approaches (*p* < 0.05, Figure 1E).

Principal component analysis showed that 65.7% of the total variance of soil properties in the five approaches could be explained by the first two principle components (PC1 and PC2) (Supplementary Figure S3). AM had higher SWC, NO_3_^−^, SOM, MBC, and MBN contents whereas NH_4_^+^ content and NH_4_^+^:NO_3_^−^ ratio were higher in NR. PM, ML, and NP had lower SWC, MBC, MBN, SOM, NO_3_^−^, NH_4_^+^, and NH_4_^+^:NO_3_^−^ ratio while higher DOC and pH (Supplementary Figure S3).

### Abundance and Composition of the Diazotrophic Community

**FIGURE 2:**
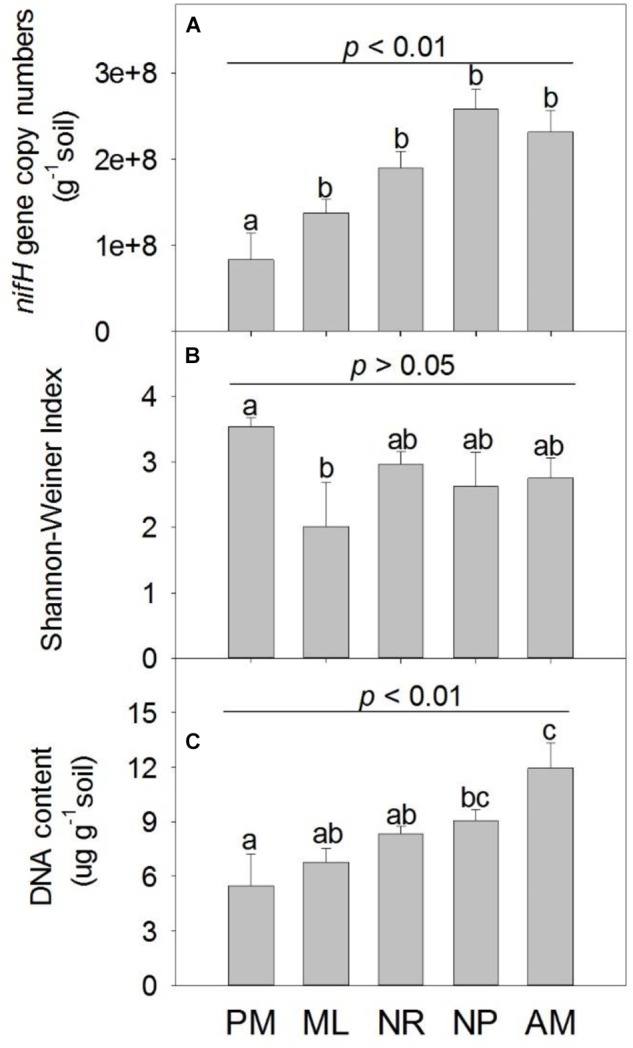
The **(A)** *nifH* gene copy numbers, **(B)** Shannon-Wiener Index of *nifH* and **(C)** soil total DNA content under five reforestation approaches in subtropical China. The five approaches were: *Pinus massoniana* monoculture (PM), mixed *Acacia crassicarpa* and *Acacia mangium* (ML), natural revegetation (NR), native tree plantation (NP), and *Acacia mangium* monoculture (AM). Error bars represent the standard errors of the mean (*n* = 6). The upper *p*-value in each chart represent the overall difference in the variation among five approaches; different lowercase letters indicate the significant differences of multiple comparisons at *p* ≤ 0.05.

**FIGURE 3:**
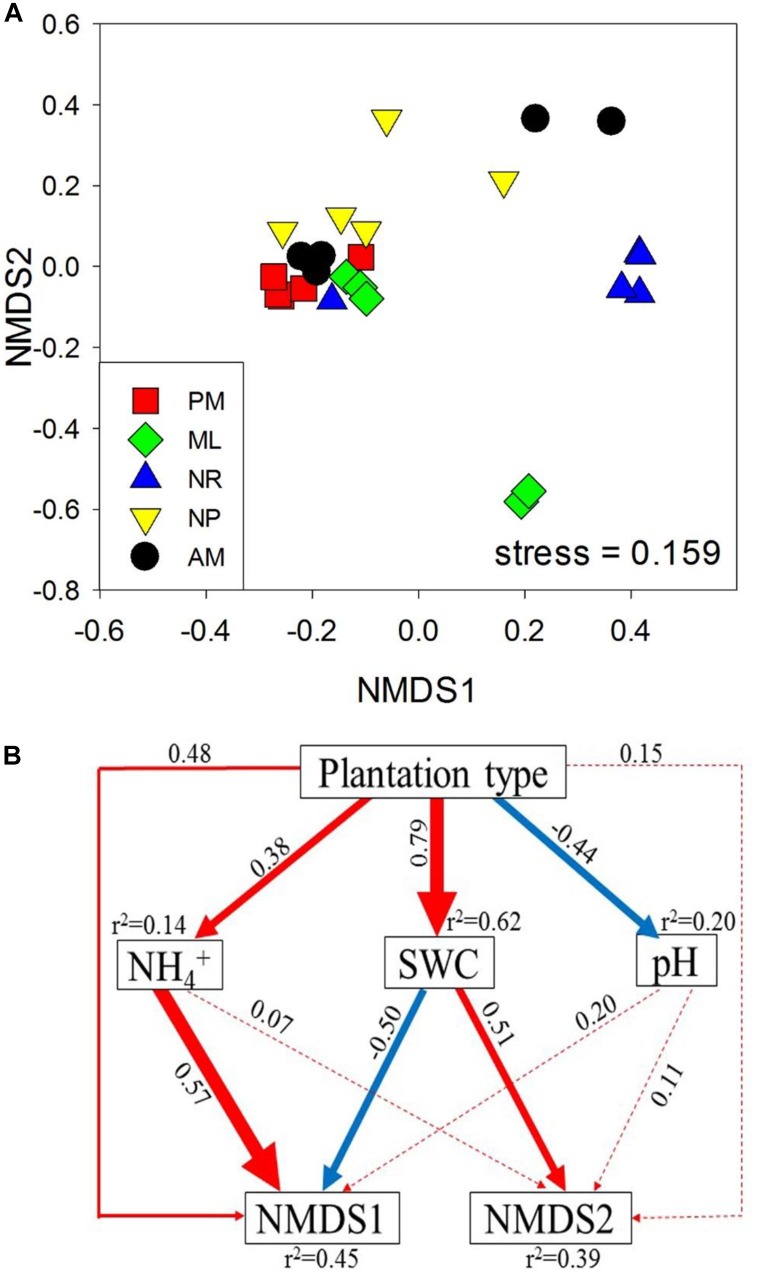
**(A)** Bray–Curtis similarity based nonmetric multidimensional scaling (NMDS) of the diazotrophic communities, determined by *nifH* amplicon sequencing, and **(B)** a structural equation model (SEM) analysis showing the potentially direct and indirect correlations between the diazotrophic community, represented by the two axes of the NMDS from **(A)**, and soil properties. Red and blue arrows indicate the positive and negative relationships, respectively. Solid and dashed arrows indicate significant and non-significant correlations, respectively, and the widths of the arrows indicate the significance levels of the correlation coefficients. Numbers adjacent to arrows are path coefficients, and the *r*^2^ values represent the explained variation of the target variables. The five approaches were: *Pinus massoniana* monoculture (PM), mixed *Acacia crassicarpa* and *Acacia mangium* (ML), natural revegetation (NR), native tree plantation (NP), and *Acacia mangium* monoculture (AM).

The *nifH* gene was most abundant in NP, followed by AM, NR, and ML (Figure 2A). PM had the lowest abundance of *nifH* compared to the other reforestation approaches, whereas the greatest Shannon-Wiener index for *nifH* was observed in PM (*p* > 0.05, Figure 2B). The total content of soil DNA was significantly higher in NP and AM than in PM (Figure 2C). The composition of diazotrophic communities differed significantly among the approaches, as indicated by the statistical significance of both the OTU abundance (*F*_4,20_ = 2.24, *p* = 0.001, Figure 3A) and OTU absence/presence (*F*_4,20_ = 2.19, *p* = 0.001, Supplementary Figure S4).

The indicator species, which contributed to the differences in community composition, were identified by an indicator species analysis. PM had 31 indicator species that were dominated by the orders *Rhizobiales* and *Clostridiales*, such as *Bradyrhizobium*, *Pleomorphomonas*, and *Ruminiclostridium*. Ten indicator species were observed in the ML, and these species were mainly distributed in the orders *Rhodospirillales* (*Azospirillum lipoferum*), *Rhizobiales* (*Azorhizobium doebereinerae*) and *Holophagales* (*Holophaga foetida*). The NR was characterized by 13 indicator species, including *Oceanospirillales*, *Rhodobacterales*, and *Corynebacteriales*. NP and AM had 7 and 10 indicator species, respectively (Supplementary Table S1).

### Associations of Diazotrophic Community Abundance and Composition With Soil Properties

**FIGURE 4:**
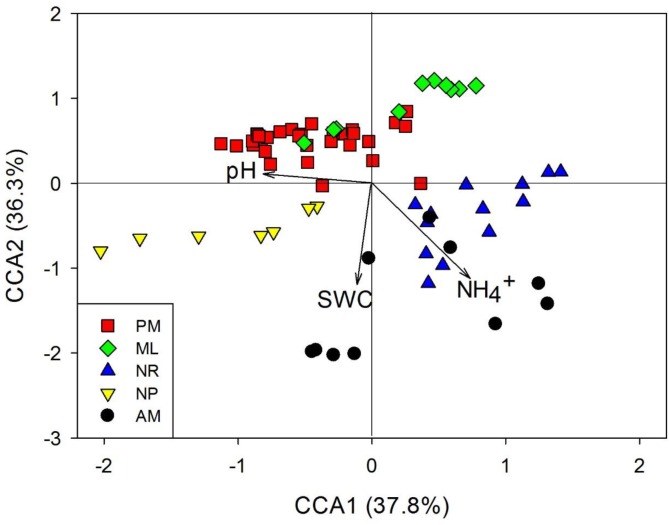
Canonical correspondence analysis (CCA) of the indicator *nifH* species composition among five reforestation approaches. The statistically significant environmental variables are presented as arrows. The five approaches were: *Pinus massoniana* monoculture (PM), mixed *Acacia crassicarpa* and *Acacia mangium* (ML), natural revegetation (NR), native tree plantation (NP), and *Acacia mangium* monoculture (AM).

The Spearman correlation, SEM and CCA were used to assess the associations between diazotrophic communities and soil physicochemical properties. Overall, *nifH* abundance and total soil DNA content were positively correlated with SWC and SOM (*p* < 0.05, Table 1), and negatively correlated with soil pH (*p* < 0.05, Table 1). Additionally, the total soil DNA content showed positive correlations to MBC and MBN (*p* < 0.05, Table 1). SEM analysis revealed that diazotrophic community composition based on the *nifH* gene was significantly correlated with NH_4_^+^ and SWC (Figure 3B). A 39–45% variation in *nifH* community composition among the reforestation approaches could be explained by the variations in vegetation type, soil NH_4_^+^, SWC, and pH. Results of CCA revealed that indicator *nifH* species among the approaches were mainly correlated with differences in soil pH, SWC, and NH_4_^+^ content (*p* < 0.05, Figure 4).

**Table 1:** Pearson correlation (r) between microbial attributes (DNA content, *nifH* abundance and Shannon-Wiener diversity) and soil physicochemical properties (SWC, pH, NH_4_^+^, NO_3_^−^, SOM, DOC, MBC, and MBN).

Variables	*NifH* gene abundance	DNA content	Shannon diversity	SWC	pH	NH_4_^+^	NO_3_^−^	SOM	DOC	MBC	MBN
*NifH* gene abundance	1	^∗∗^	NS	^∗^	^∗^	NS	NS	^∗^	NS	NS	NS
DNA content	0.74	1	NS	^∗∗^	^∗^	NS	NS	^∗∗^	NS	^∗∗^	^∗^
Shannon diversity	−0.32	−0.14	1	NS	NS	NS	NS	NS	NS	NS	NS
SWC	0.45	0.71	0.01	1	^∗^	NS	NS	^∗^	^∗^	^∗∗^	^∗∗^
pH	−0.45	−0.40	−0.01	−0.43	1	NS	NS	^∗∗^	NS	NS	NS
NH_4_^+^	0.25	0.27	0.01	0.13	−0.19	1	^∗∗^	^∗∗^	NS	NS	NS
NO_3_^−^	0.03	0.11	0.05	0.14	−0.28	0.55	1	^∗^	NS	NS	NS
SOM	0.47	0.61	0.07	0.50	−0.61	0.59	0.43	1	NS	^∗^	NS
DOC	0.12	0.14	−0.13	0.45	−0.35	−0.25	0.21	0.24	1	NS	NS
MBC	0.32	0.60	−0.08	0.80	−0.32	0.17	0.25	0.40	0.39	1	^∗∗^
MBN	0.26	0.49	−0.20	0.71	−0.22	0.22	0.14	0.20	0.24	0.88	1

*Data from all the five reforestation approaches were used in the analysis (n = 30). SWC, soil water content (%); SOM, soil organic matter (g kg^−1^); DOC, dissolved organic carbon (mg C kg^−1^); MBC, microbial biomass carbon (mg C kg^−1^); MBN, microbial biomass nitrogen (mg N kg^−1xs^). ^∗∗^p ≤ 0.01; ^∗^p ≤ 0.05.*

### OPU Analyses

**FIGURE 5:**
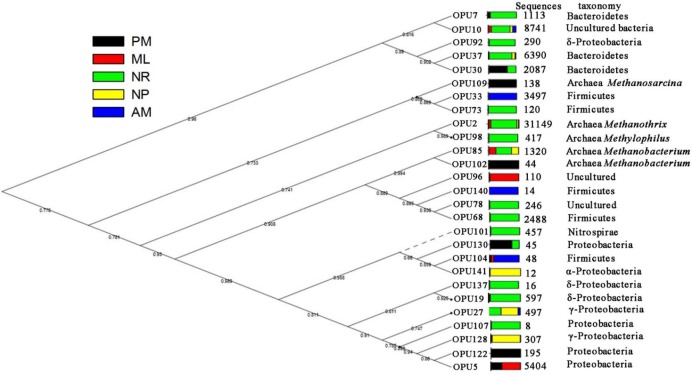
Maximum likelihood phylogenetic tree of translated amino acid sequences of the *nifH* gene in five reforestation approaches: black, *Pinus massoniana* monoculture plantation (PM); red, mixed *Acacia crassicarpa* and *Acacia mangium* (ML); green, natural revegetation (NR); yellow, native tree plantation (NP); and blue, *Acacia mangium* monoculture plantation (AM). Only the operational protein units (OPU) containing more than five sequences are displayed. The bootstrap support values are presented on the respective branches, and the dashed branches represent bootstrap support values less than 70%. The number of sequences and the taxonomic affiliations of the OPUs are indicated on the right.

The *nifH* sequences were translated into amino acid sequences and then aligned on the functional gene pipeline (Fungene) within the Ribosomal Data Project to cluster the OPUs. The OPU identification and phylogenetic clustering of the translated *nifH* sequences were used to investigate the responses of the diazotrophic community to different reforestation approaches at the protein level. A total of 150 OPUs were identified based on the 90% similarity cutoff for translated amino acid sequences. These OPUs contained more translated amino acid sequences (80%) than those identified based on other similarity cutoffs, such as 99%, 97%, 95%, and 80%. Consequently, we only focused on the OPUs from the 90% similarity cutoff. A total of 27 OPUs containing more than five sequences each were used to construct the phylogenetic tree (Figure 5). Among the 27 OPUs, more than 50% of the sequences were clustered into OPUs belonging to Archaea (OPU2, OPU85, OPU98, OPU102, and OPU109), followed by the OPUs related to *Bacteroidetes* (15%), *Proteobacteria* (11%), and *Firmicutes* (9%). Two OPUs containing 14% of the sequences (OPU10 and OPU78) were similar to the sequences of uncultured microorganisms. The distributions of these OPUs varied substantially among the five reforestation approaches. For example, OPUs assigned to Archaea (OPU2, OPU85, and OPU98), *Bacteroidetes* (OPU37), and δ-*Proteobacteria* (OPU19, OPU92, and OPU137) were more commonly found in NR (83%, 73%, and 96% of the total sequences in Archaea, *Bacteroidetes*, and δ-*Proteobacteria* were from NR). Three OPUs (OPU27, OPU128, and OPU141; most closely related to α- and γ-*Proteobacteria*) were more commonly found in NP (100% and 70% of the total sequences in α- and γ-*Proteobacteria* were from NP). OPU33 and OPU140 (affiliated with *Firmicutes*) were only detected in AM, and OPU96 only presented in ML. Five OPUs (OPU30, OPU102, OPU109, OPU122, and OPU130; most closely related to *Bacteroidetes*, Archaea, and *Proteobacteria*) were more commonly found in PM.

## Discussion

### Diazotrophic Community Abundance and Relating Soil Properties

We investigated the abundance and composition of the diazotrophic community by quantifying and sequencing the *nifH* gene in bulk soil to compare the responses of free-living N-fixers to different reforestation approaches and to study their relation to soil N availability. Previous studies, focused on the 16S rRNA gene or phospholipid fatty acid (PLFA), implied significant shifts in the microbial community composition, and diversity in response to changes in vegetation during forest restoration (Banning et al., 2011; Song et al., 2015; Strickland et al., 2017). However, responses of the functional microorganisms involved in nutrient cycling have received little attention. The diazotrophic microorganisms are primary drivers of soil BNF that may be significantly correlated with diazotrophic community attributes, including abundance, composition, and activity (Bowers et al., 2008; Reed et al., 2010; Pogoreutz et al., 2017). In this study, both the abundance and composition of the diazotrophic community varied significantly among the reforestation approaches, which may indicate variation in the BNF rate. In the diazotrophic community, the gene *nifH* encodes a subunit of nitrogenase that is necessary for the catalysis of microbial N_2_-fixation. Thus, the *nifH* abundance might be related to BNF capacity (Newton, 2013). Overall, the average abundance of *nifH* in the reforestation approaches evaluated was higher than in other natural forest soils (Hayden et al., 2010; Mirza et al., 2014). This is possibly related to the limitation in soil N at the early stages of forest recovery (Kamijo et al., 2002; Zhan et al., 2009). In our study, soil inorganic N contents correlated with the *nifH* community composition but not with *nifH* abundance, suggesting that the composition of the diazotrophic community may be more critical than abundance in affecting soil N availability.

The positive correlation between *nifH* abundance and SOM indicated a covariation between diazotrophic abundance and the organic C content. A greater organic C content could support more diazotrophs, possibly because the BNF conducted by diazotrophs is energy consuming (Klucas, 1991). In addition to SOM, greater SWC may indirectly lead to a greater availability of organic C substrate to diazotrophs by enhancing the diffusive transport of organic soluble substrates and the mobility of microorganisms (Borken and Matzner, 2009; Ding et al., 2015). In line with this, in our study *nifH* abundance correlated positively with SWC. The greater *nifH* abundance in NP compared to the other reforestation approaches may have been related to the greatest SWC in the NP. Soils in the AM had the highest SOM and SWC and lowest pH. However, the *nifH* gene was not the most abundan in the AM, which may result from the effects of other soil properties, including phosphorus content (Orchard et al., 2009), soil texture and aggregate size (Poly et al., 2001).

### Diazotrophic Community Composition and Its Correlation With Soil N Content

The SWC and NH_4_^+^ contributed most to the differences in soil environments among reforestation approaches, correlating significantly with the diazotrophic community composition and indicator species. The AM and NR had particularly higher NH_4_^+^ content compared to the other reforestation approaches, with correlations between the indicator species and the high content of NH_4_^+^. The indicator species compositions in the PM and ML were mainly associated with the low SWC. In addition, OPU2, OPU85, and OPU98 affiliated with *Methanothrix*, *Methylophilus*, and *Methanobacterium*, respectively, were common in NR. Although the coupling of methanogenesis and BNF processes remained unclear, the ability of methanogenic archaea to fix N_2_, and the interlinkage between C and N cycles have been well studied (Belay et al., 1984; Murray and Zinder, 1984). Our results suggest that archaeal biological N_2_ fixation was important and potentially associated with C cycling in NR. The AM had higher inorganic N (NH_4_^+^ and NO_3_^−^) and MBN than other reforestation approaches, suggesting that high soil inorganic N content could have driven microbial N immobilization in AM but not in NR. Possibly, the low soil organic C content in NR resulted in less microbial growth and microbial N immobilization. However, under ML, the diazotrophic community composition changed substantially, and the soil N content decreased markedly when compared to AM. The majority of indicator species in the PM were *Rhizobiales*. The dominance of *Rhizobiales* in PM may relate to the low quality of organic C resources owing to recalcitrant litter (Zhang et al., 2008; Kirschbaum, 2013; Wang et al., 2018). Planting some dominant native species during the initial forest restoration may be helpful in the functional and structural recovery of degraded forest ecosystems (Lu et al., 2017). In this study, the NP was dominated by versatile diazotrophic groups, such as *Paraburkholderia*, *Candidatus Koribacter*, and *Desulfospira*, which could help the plant resist diseases (Rahman et al., 2018), and tolerate soil acidification and infertility (Kai et al., 1997) during the early stages of restoration.

## Conclusion

Our study demonstrated that the abundance and composition of the diazotrophic community varied among different reforestation approaches implemented in subtropical China. The variations of diazotrophic community were closely associated with soil pH, SWC, NH_4_^+^, and SOM. However, soil NH_4_^+^ was significantly correlated with the composition of diazotrophic community and not the abundance, suggesting that the composition may have a greater effect on soil N availability. This study suggested that determining the diazotrophic community composition during the early stages of reforestation can help us choose effective pioneer species for promoting soil N supply and to understand the mechanisms involved in microbial communities.

## Author Contributions

JC and WS conceived the research. JC performed the experiments and analyzed the data. JC, WS, HX, YL, and TL wrote and edited the manuscript.

## Conflict of Interest Statement

The authors declare that the research was conducted in the absence of any commercial or financial relationships that could be construed as a potential conflict of interest.

## Footnotes

1 http://progenomes.embl.de/
