# Supplementary material for: The Composition of Nitrogen-Fixing Microorganisms Correlates With Soil Nitrogen Content During Reforestation: A Comparison Between Legume and Non-legume Plantations
Source: Front Microbiol. 2019 Mar 14;10:508. doi: 10.3389/fmicb.2019.00508 (PMC6427063; doi:10.3389/fmicb.2019.00508)
Supplement: Supplementary file 1 [file Data_Sheet_1.docx]

**The Composition of Nitrogen-Fixing Microorganisms Correlates with Soil Nitrogen Content during Reforestation: A Comparison between Legume and Non-legume Plantations**

**Running head:** N-fixer composition affects N content

Jie Chen^1^, Weijun Shen^2^, Han Xu^1^, Yide Li^1^, Tushou Luo^1^

**^1^** Research Institute of Tropical Forestry, Chinese Academy of Forestry, Longdong, Guangzhou, 510520, P.R. China

^2^ **Key Laboratory of Vegetation Restoration and Management of Degraded Ecosystems, South China Botanical Garden, Chinese Academy of Sciences, 7**23 Xinke Rd. Tianhe District, Guangzhou 510650, PR China

*Corresponding author: Dr. Jie Chen

Tel: + 86 20 8703 2619

Fax: +86 20 8703 1622

E-mail: chenjiecaf@hotmail.com


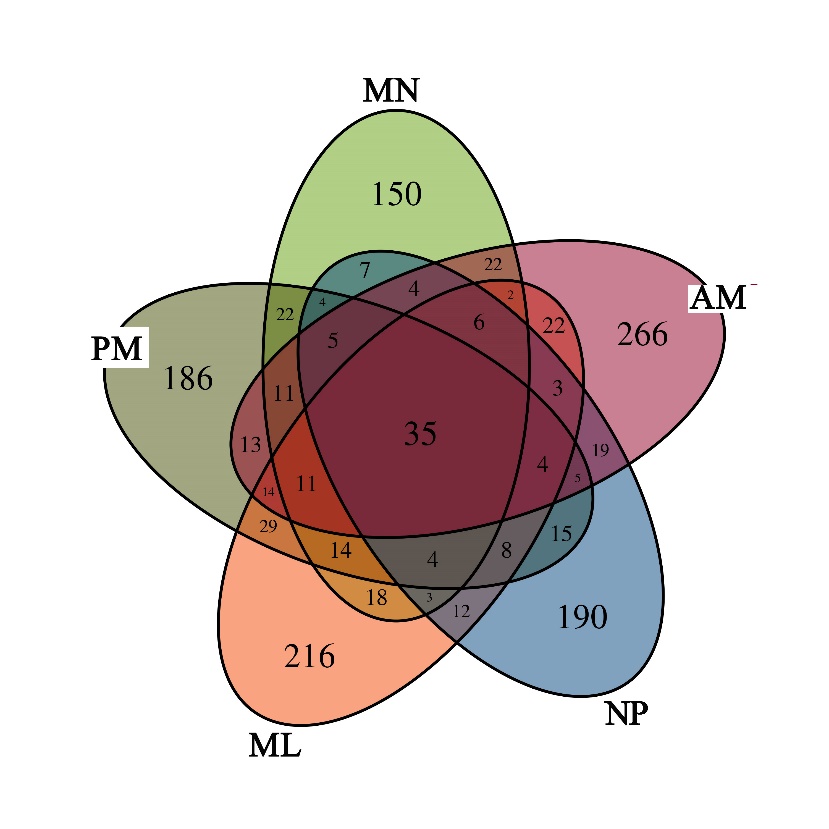


**Figure S1** Venn analysis showing the shared and specialized diazotrophic species among the five reforestation stands: *pinus massoniana* monoculture plantation (PM), mixed *Acacia crassicarpa* and *Acacia mangium* plantation (mixed legumes, ML), natural revegetation (NR), native tree plantation (NP), *Acacia mangium* monoculture plantation (AM).


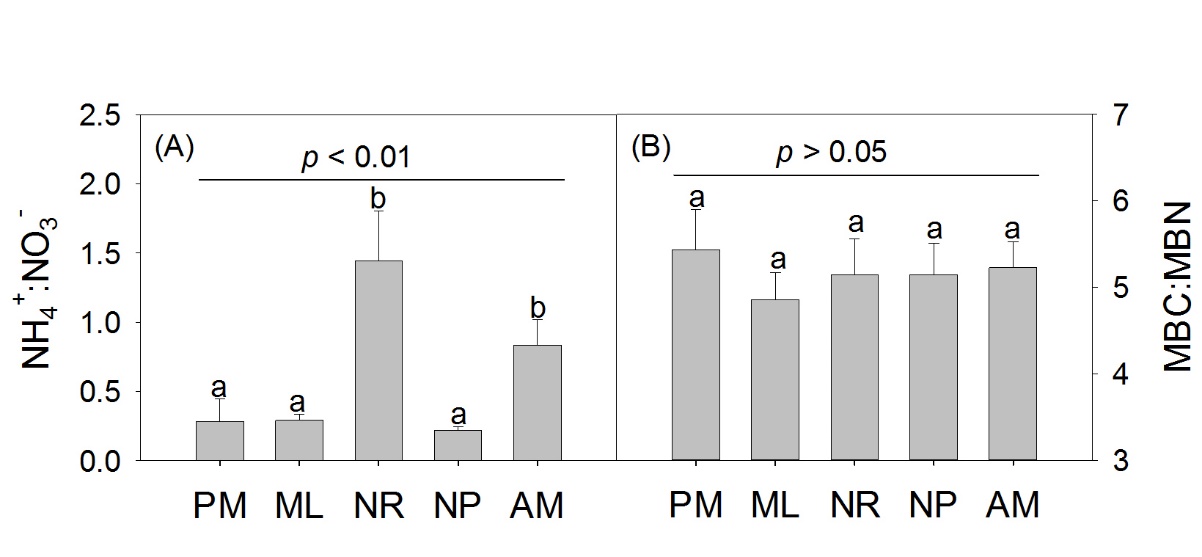


**Figure S2** Ratios of (A) NH_4_^+^: NO_3_^-^ and (B) MBC: MBN in response to different forest restoration approaches in subtropical region of South China: *pinus massoniana* monoculture plantation (PM), mixed *Acacia crassicarpa* and *Acacia mangium* (mixed legumes, ML), natural revegetation (NR), native tree plantation (NP), *Acacia mangium* monoculture plantation (AM). Gray bars represent the average value of six samples, and the error bars represent the standard errors of the mean. The upper *p* value in each chart represent the overall difference of the variation among five approaches, different lowercase stand for the significant difference from multiple comparisons with *p* ≤ 0.05.


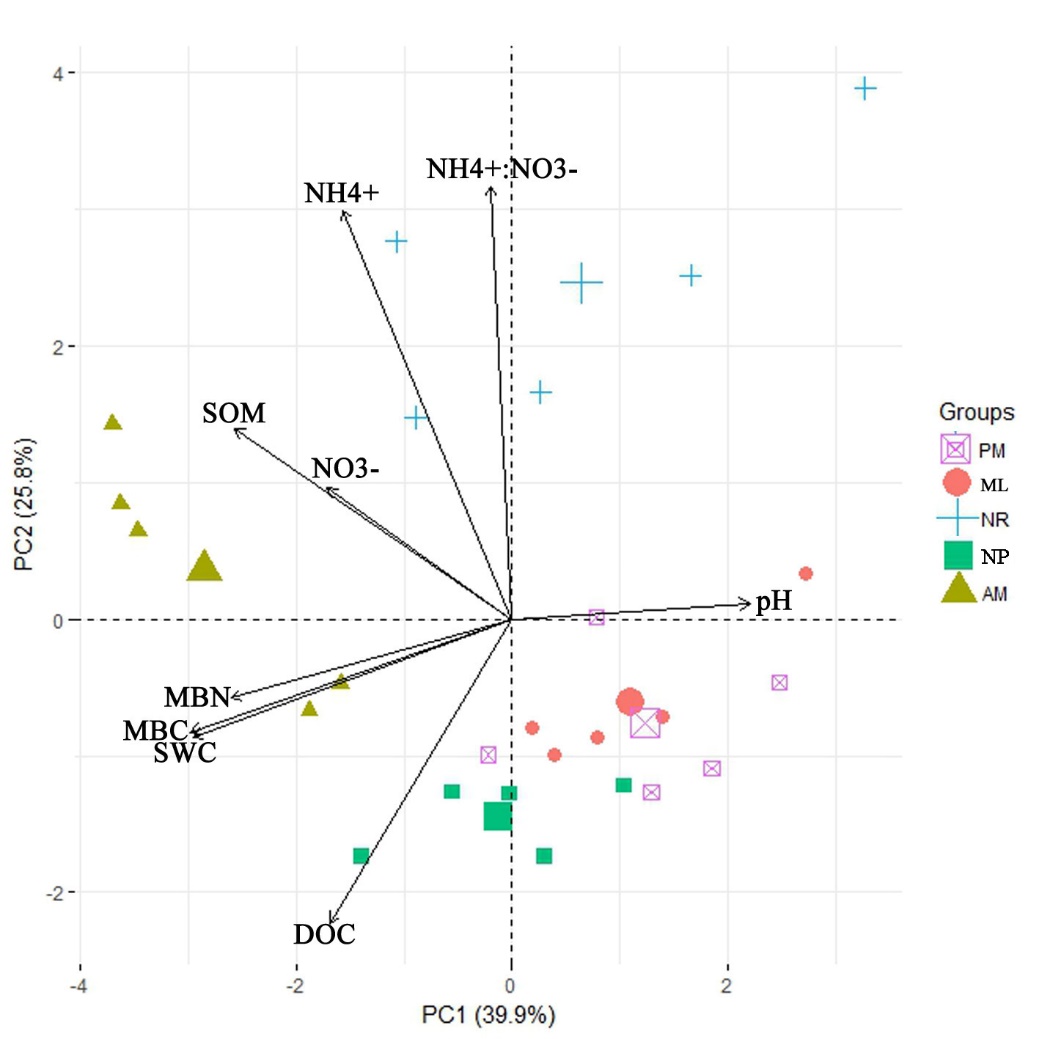


**Figure S3** Principle component analysis of the soil properties across the five restoration stands. See figure S1 for the information of the abbreviations.


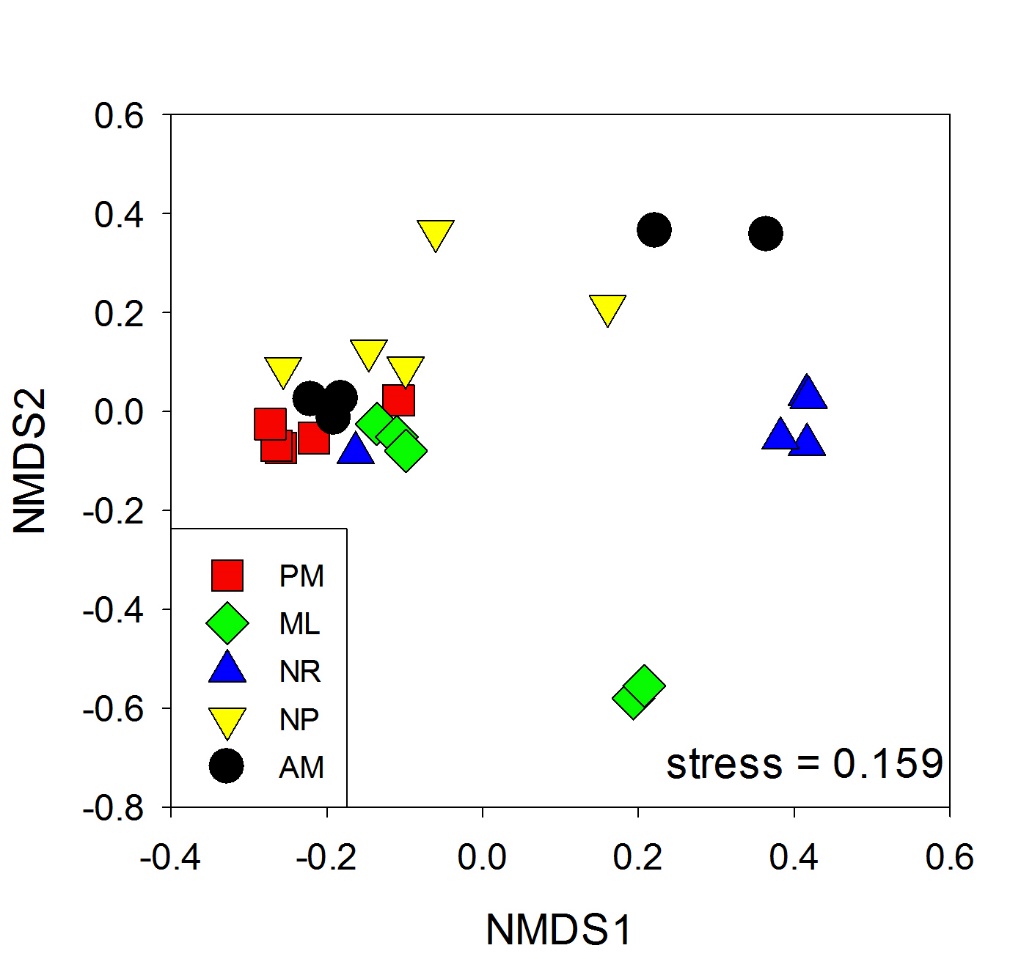


**Figure S4** Non-metric multidimensional scaling (NMDS) analysis of the *nifH* gene structure based on the Sørensen similarity index. See Figure S1 for the information of the abbreviations.

**Table S1** Taxonomy of the indicator species of the diazotrophic community under each of the five restoration approaches: *pinus massoniana* monoculture plantation (PM), mixed *Acacia crassicarpa* and *Acacia mangium* (thereafter mixed legumes, ML), natural revegetation (NR), native tree plantation (NP), *Acacia mangium* monoculture plantation (AM).

| Group | Indicator  value | probability | OTU | Kingdom | Phylum | Class | Order | Family | Genus | Species |
| --- | --- | --- | --- | --- | --- | --- | --- | --- | --- | --- |
| PM | 0.8511 | 0.002 | OTU103 | Bacteria | Verrucomicrobia | Opitutales | Opitutae | Opitutaceae |  |  |
| PM | 0.6 | 0.022 | OTU1108 | Bacteria | Firmicutes | Clostridiales | Clostridia |  |  |  |
| PM | 1 | 0.001 | OTU113 | Bacteria | Proteobacteria | Sphingomonadales | Alphaproteobacteria | Sphingomonadaceae | Novosphingobium | Novosphingobium sp. Rr 2-17 |
| PM | 0.7033 | 0.001 | OTU14 | Bacteria | Proteobacteria | Rhizobiales | Alphaproteobacteria |  |  |  |
| PM | 0.5987 | 0.018 | OTU152 | Bacteria | Proteobacteria | Syntrophobacterales | Deltaproteobacteria | Syntrophaceae | Desulfobacca | Desulfobacca acetoxidans |
| PM | 0.6859 | 0.017 | OTU158 | Bacteria | Proteobacteria |  | Deltaproteobacteria |  |  |  |
| PM | 0.8412 | 0.007 | OTU165 | Bacteria | Firmicutes | Clostridiales | Clostridia | Ruminococcaceae | Ruminiclostridium | [Clostridium] termitidis |
| PM | 0.8517 | 0.002 | OTU167 | Bacteria | Proteobacteria | Rhizobiales | Alphaproteobacteria |  |  |  |
| PM | 0.5947 | 0.026 | OTU206 | Unknown |  |  |  |  |  |  |
| PM | 0.5971 | 0.035 | OTU226 | Unknown |  |  |  |  |  |  |
| PM | 0.7225 | 0.001 | OTU26 | Bacteria | Chlorobi | Chlorobiales | Chlorobia | Chlorobiaceae |  |  |
| PM | 0.8 | 0.002 | OTU282 | Bacteria | Firmicutes |  |  |  |  |  |
| PM | 0.7583 | 0.002 | OTU283 | Bacteria | Bacteroidetes | Marinilabiliales | Bacteroidia | Marinilabiliaceae | Saccharicrinis | Saccharicrinis fermentans |
| PM | 0.7499 | 0.005 | OTU39 | Bacteria | Proteobacteria |  |  |  |  |  |
| PM | 0.5799 | 0.021 | OTU390 | Bacteria | Proteobacteria |  | Alphaproteobacteria |  |  |  |
| PM | 0.7963 | 0.002 | OTU391 | Bacteria | Actinobacteria | Corynebacteriales | Actinobacteria | Mycobacteriaceae | Mycobacterium | Mycobacterium tusciae |
| PM | 0.5458 | 0.034 | OTU42 | Bacteria | Proteobacteria | Desulfovibrionales | Deltaproteobacteria | Desulfovibrionaceae | Desulfovibrio |  |
| PM | 0.5822 | 0.02 | OTU434 | Bacteria | Proteobacteria | Desulfobacterales | Deltaproteobacteria | Desulfobacteraceae | Desulfatibacillum | Desulfatibacillum alkenivorans |
| PM | 0.6 | 0.025 | OTU481 | Bacteria | Proteobacteria | Rhizobiales | Alphaproteobacteria |  |  |  |
| PM | 0.5847 | 0.022 | OTU482 | Bacteria | Proteobacteria |  | Deltaproteobacteria |  |  |  |
| PM | 0.6 | 0.032 | OTU495 | Bacteria | Proteobacteria | Rhizobiales | Alphaproteobacteria | Bradyrhizobiaceae | Bradyrhizobium | Bradyrhizobium sp. DOA9 |
| PM | 0.595 | 0.024 | OTU513 | Bacteria | Proteobacteria | Rhizobiales | Alphaproteobacteria | Bradyrhizobiaceae | Bradyrhizobium | Bradyrhizobium sp. DOA9 |
| PM | 0.4261 | 0.04 | OTU525 | Bacteria | Proteobacteria | Rhizobiales | Alphaproteobacteria | Methylocystaceae | Pleomorphomonas | Pleomorphomonas oryzae |
| PM | 0.569 | 0.041 | OTU726 | Bacteria |  |  |  |  |  |  |
| PM | 0.7333 | 0.012 | OTU75 | Bacteria | Firmicutes | Clostridiales | Clostridia |  |  |  |
| PM | 0.4536 | 0.038 | OTU78 | Bacteria | Verrucomicrobia | Opitutales | Opitutae | Opitutaceae |  |  |
| PM | 0.5172 | 0.047 | OTU79 | Unknown |  |  |  |  |  |  |
| PM | 0.9189 | 0.001 | OTU871 | Unknown |  |  |  |  |  |  |
| PM | 0.5983 | 0.014 | OTU94 | Bacteria | Firmicutes |  |  |  |  |  |
| PM | 0.6 | 0.019 | OTU955 | Unknown |  |  |  |  |  |  |
| PM | 0.5743 | 0.034 | OTU97 | Unknown |  |  |  |  |  |  |
| ML | 0.6 | 0.017 | OTU1297 | Unknown |  |  |  |  |  |  |
| ML | 0.5965 | 0.027 | OTU146 | Bacteria | Proteobacteria | Rhodospirillales | Alphaproteobacteria | Rhodospirillaceae | Azospirillum | Azospirillum lipoferum |
| ML | 0.6 | 0.025 | OTU20 | Bacteria | Proteobacteria |  | Betaproteobacteria |  |  |  |
| ML | 0.5876 | 0.033 | OTU29 | Bacteria | Proteobacteria | Rhizobiales | Alphaproteobacteria | Xanthobacteraceae | Azorhizobium | Azorhizobium doebereinerae |
| ML | 0.6 | 0.028 | OTU332 | Bacteria | Proteobacteria |  | Alphaproteobacteria |  |  |  |
| ML | 0.5589 | 0.033 | OTU338 | Bacteria | Acidobacteria | Holophagales | Holophagae | Holophagaceae | Holophaga | Holophaga foetida |
| ML | 0.6204 | 0.009 | OTU665 | Unknown |  |  |  |  |  |  |
| ML | 0.5962 | 0.02 | OTU74 | Bacteria |  |  |  |  |  |  |
| ML | 0.5889 | 0.033 | OTU753 | Bacteria | Proteobacteria |  | Alphaproteobacteria |  |  |  |
| ML | 0.599 | 0.022 | OTU96 | Bacteria | Proteobacteria |  |  |  |  |  |
| NR | 0.8 | 0.001 | OTU1124 | Bacteria |  |  |  |  |  |  |
| NR | 0.5131 | 0.02 | OTU13 | Bacteria | Proteobacteria | Oceanospirillales | Gammaproteobacteria | Halomonadaceae | Halomonas | Halomonas stevensii |
| NR | 0.6445 | 0.003 | OTU222 | Bacteria |  |  |  |  |  |  |
| NR | 0.6 | 0.025 | OTU256 | Bacteria | Proteobacteria | Rhizobiales | Alphaproteobacteria | Phyllobacteriaceae | Mesorhizobium |  |
| NR | 0.724 | 0.008 | OTU301 | Bacteria | Proteobacteria | Rhodobacterales | Alphaproteobacteria | Rhodobacteraceae | Roseovarius | Roseovarius nubinhibens |
| NR | 0.6 | 0.019 | OTU346 | Unknown |  |  |  |  |  |  |
| NR | 0.5779 | 0.027 | OTU354 | Bacteria |  |  |  |  |  |  |
| NR | 0.8 | 0.008 | OTU49 | Unknown |  |  |  |  |  |  |
| NR | 0.6 | 0.023 | OTU499 | Bacteria | Actinobacteria | Corynebacteriales | Actinobacteria | Nocardiaceae | Rhodococcus | Rhodococcus defluvii |
| NR | 0.7924 | 0.004 | OTU502 | Bacteria | Bacteroidetes | Cytophagales | Cytophagia | Flammeovirgaceae | Nafulsella | Nafulsella turpanensis |
| NR | 0.6 | 0.022 | OTU595 | Bacteria | Proteobacteria | Campylobacterales | Epsilonproteobacteria | Helicobacteraceae | Helicobacter | Helicobacter saguini |
| NR | 0.4571 | 0.034 | OTU752 | Unknown |  |  |  |  |  |  |
| NR | 0.6 | 0.019 | OTU832 | Unknown |  |  |  |  |  |  |
| NP | 0.6746 | 0.022 | OTU15 | Bacteria |  |  |  |  |  |  |
| NP | 0.5765 | 0.045 | OTU196 | Bacteria | Proteobacteria | Rhizobiales | Alphaproteobacteria |  |  |  |
| NP | 0.6 | 0.026 | OTU223 | Bacteria | Proteobacteria | Desulfobacterales | Deltaproteobacteria | Desulfobacteraceae | Desulfospira | Desulfospira joergensenii |
| NP | 0.5919 | 0.042 | OTU244 | Bacteria | Verrucomicrobia | Opitutales | Opitutae | Opitutaceae |  |  |
| NP | 0.5859 | 0.036 | OTU248 | Unknown |  |  |  |  |  |  |
| NP | 0.5368 | 0.045 | OTU567 | Bacteria | Acidobacteria | Acidobacteriales | Acidobacteriia | Acidobacteriaceae | Candidatus Koribacter | Candidatus Koribacter versatilis |
| NP | 0.7944 | 0.006 | OTU70 | Bacteria | Proteobacteria | Burkholderiales | Betaproteobacteria | Burkholderiaceae | Paraburkholderia | Paraburkholderia kururiensis |
| AM | 0.5333 | 0.047 | OTU1203 | Bacteria |  |  |  |  |  |  |
| AM | 1 | 0.001 | OTU296 | Bacteria | Actinobacteria |  | Actinobacteria |  |  |  |
| AM | 0.8168 | 0.003 | OTU30 | Bacteria | Proteobacteria | Rhodospirillales | Alphaproteobacteria | Rhodospirillaceae | Rhodospirillum | Rhodospirillum rubrum |
| AM | 0.6 | 0.026 | OTU367 | Bacteria | Actinobacteria | Streptomycetales | Actinobacteria | Streptomycetaceae | Streptomyces | Streptomyces sclerotialus |
| AM | 0.791 | 0.003 | OTU445 | Bacteria | Firmicutes | Bacillales | Bacilli | Alicyclobacillaceae | Alicyclobacillus | Alicyclobacillus contaminans |
| AM | 0.6 | 0.017 | OTU542 | Unknown |  |  |  |  |  |  |
| AM | 0.7981 | 0.006 | OTU55 | Bacteria |  |  |  |  | Thermobaculum | Thermobaculum terrenum |
| AM | 0.5114 | 0.045 | OTU6 | Bacteria | Proteobacteria | Oceanospirillales | Gammaproteobacteria | Halomonadaceae | Halomonas | Halomonas stevensii |
| AM | 0.5731 | 0.024 | OTU614 | Bacteria | Acidobacteria | Acidobacteriales | Acidobacteriia | Acidobacteriaceae | Candidatus Koribacter | Candidatus Koribacter versatilis |
| AM | 0.6 | 0.017 | OTU857 | Bacteria | Firmicutes | Bacillales | Bacilli | Bacillaceae | Bacillus | Bacillus subtilis |
